# Serplulimab, a novel anti-PD-1 antibody, in patients with microsatellite instability-high solid tumours: an open-label, single-arm, multicentre, phase II trial

**DOI:** 10.1038/s41416-022-02001-3

**Published:** 2022-10-19

**Authors:** Shukui Qin, Jin Li, Haijun Zhong, Chuan Jin, Lili Chen, Xianglin Yuan, Qingxia Fan, Kehe Chen, Peiguo Cao, Jianjun Xiao, Da Jiang, Tao Zhang, Hongyu Zhang, Xicheng Wang, Wei Wang, Lin Han, Qingyu Wang, Jun Zhu, the Serplulimab-MSI-H Investigators

**Affiliations:** 1 Department of Oncology, Qinhuai Medical Area, Eastern Theater General Hospital of PLA China, Nanjing, China; 2 Department of Oncology, Tongji University Shanghai East Hospital, Shanghai, China; 3 Department of Abdominal Oncology, Cancer Hospital of the University of Chinese Academy of Sciences (Zhejiang Cancer Hospital), Hangzhou, China; 4 Department of Oncology, Affiliated Cancer Hospital and Institute of Guangzhou Medical University, Guangzhou, China; 5 Department of Hematology and Oncology, Taizhou First People’s Hospital, Taizhou, China; 6 Department of Oncology, Tongji Hospital, Huazhong University of Science and Technology, Wuhan, China; 7 Department of Oncology, The First Affiliated Hospital of Zhengzhou University, Zhengzhou, China; 8 Department of Oncology, The People’s Hospital of Guangxi Zhuang Autonomous Region, Nanning, China; 9 Department of Oncology, The Third Xiangya Hospital of Central South University, Changsha, China; 10 Chemotherapeutic Department, Zhongshan City People’s Hospital, Zhongshan, China; 11 Department of Oncology, The Fourth Hospital of Hebei Medical University, Shijiazhuang, China; 12 Department of Oncology, The First Affiliated Hospital of Chongqing Medical University, Chongqing, China; 13 Cancer Center, The Fifth Affiliated Hospital Sun Yat-sen University, Zhuhai, China; 14 Department of Oncology, The First Affiliated Hospital of Guangdong Pharmaceutical University, Guangzhou, China; 15 Department of Gastrointestinal Oncology, The First People’s Hospital of Foshan, Foshan, China; 16 Shanghai Henlius Biotech, Inc., Shanghai, China

**Keywords:** Cancer therapy, Biomarkers, Phase II trials, Immunotherapy

## Abstract

**Background:**

Microsatellite instability-high/mismatch repair-deficient (MSI-H/dMMR) tumours have a high response rate to immunotherapy. Antitumour activity and safety of serplulimab, a novel humanised anti-PD-1 monoclonal antibody, were evaluated in this phase II study.

**Methods:**

In this ongoing, single-arm, open-label, phase II trial, patients with previously treated unresectable or metastatic MSI-H/dMMR solid tumours received intravenous serplulimab 3 mg/kg every 2 weeks for up to 52 cycles. The primary endpoint was objective response rate (ORR) assessed by an independent radiological review committee per Response Evaluation Criteria in Solid Tumors v1.1. Secondary endpoints included additional efficacy measures, safety, and tolerability.

**Results:**

As of 9 January 2021, 108 patients were enrolled, and 68 patients with confirmed MSI-H solid tumours were included in the main efficacy analysis population (MEAP). The median follow-up duration in the MEAP was 7.7 months, with an ORR of 38.2% (95% confidence interval, 26.7–50.8). Of the 108 patients, grade ≥3 treatment-emergent adverse events were reported in 53 (49.1%) patients; immune-related adverse events occurred in 52 (48.1%) patients.

**Conclusions:**

Serplulimab demonstrates a durable antitumour effect and a manageable safety profile in previously treated patients with MSI-H solid tumours. Serplulimab is a promising tissue-agnostic treatment for previously treated MSI-H solid tumours.

**Trial registration:**

NCT03941574.

## Background

Patients with metastatic microsatellite instability-high (MSI-H) or mismatch repair-deficient (dMMR) tumours represent a distinct patient population. Depending on the type and stage of cancer, MSI-H/dMMR patients may have a better (e.g. MSI-H stage I/II colorectal cancer [CRC], gastric cancer, or bladder cancer) or worse prognosis (e.g. MSI-H stage III CRC or breast cancer) and might respond poorly to chemotherapy compared to patients with microsatellite stable/microsatellite instability-low (MSS/MSI-L) tumours [1–5]. MSI-H accounts for <5–33% of tumours, and a meta-analysis showed that the frequencies were similar between Chinese and Western populations [6]. The burden of MSI-H cancers is estimated to be more than 1 million and about 0.3 million new cases per year worldwide and in China, respectively [6–10].

MSI-H/dMMR tumours express a large array of neoantigens due to a high level of mutations [11, 12], and their microenvironment is characterised by immune cell infiltration, coupled with an upregulation of immune checkpoint proteins in tumour cells [13]. These establish the biological rationale of immune checkpoint blockade for MSI-H/dMMR tumours. The durable and encouraging tumour response observed with pembrolizumab (objective response rate [ORR] of 39.6%) across MSI-H/dMMR tumours provides evidence for the clinical benefits of using immunotherapy [14]. Pembrolizumab received tissue-agnostic approvals for MSI-H/dMMR tumours, which marked an advancement in precision medicine [15, 16]. Nivolumab also received an approval based on MSI-H/dMMR, but only in previously treated CRC [17]. Currently, patients with MSI-H/dMMR tumours have limited treatment options, and thus alternative therapeutic agents may be beneficial.

Serplulimab (HLX10) is a novel humanised monoclonal anti-PD-1 antibody. In a phase I study involving patients with previously treated advanced or metastatic solid tumours (NCT03468751), serplulimab up to 10 mg/kg was safe and well tolerated [18]. Here we present data from a phase II study evaluating the antitumour activity and safety of serplulimab 3 mg/kg in Chinese patients with unresectable or metastatic MSI-H/dMMR solid tumours in the subsequent line setting.

## Methods

### Study design

This ongoing, single-arm, open-label, phase II trial was conducted at 39 study sites in China (of which 33 enrolled patients). Patients received intravenous serplulimab 3 mg/kg every 2 weeks for up to 52 cycles or until loss of clinical benefit, unacceptable toxicity, death, or withdrawal of consent. Patients could continue serplulimab treatment after a first documented disease progression (PD) per Response Evaluation Criteria in Solid Tumors (RECIST) v1.1 [19] if it was neither symptomatic nor rapidly progressive requiring urgent intervention, and if their Eastern Cooperative Oncology Group performance status did not deteriorate. Subsequent treatment for patients with confirmed PD at the next assessment (≥4 weeks from the first documented PD) by response criteria for cancer immunotherapy trials (iRECIST) [20] was at the discretion of the investigator. The trial was registered with ClinicalTrials.gov (NCT03941574).

### Patients

Eligible patients were 18–75 years of age, had histologically or cytologically confirmed unresectable or metastatic MSI-H/dMMR solid tumours as assessed at central laboratory or study sites, and had progressed on or were intolerant to at least one prior line of standard therapy. Previous systemic antitumour therapy must have been discontinued ≥2 weeks prior to study treatment, and adverse events (AEs) must have resolved to at least grade 1 (graded per National Cancer Institute Common Terminology Criteria for Adverse Events v5.0, with the exception of grade 2 alopecia). Patients must have at least one measurable lesion as per RECIST v1.1, an Eastern Cooperative Oncology Group performance status score of 0 or 1 within 7 days before initiating study treatment, adequate organ function, and life expectancy of ≥12 weeks. Details of the inclusion and exclusion criteria are provided in Supplementary Material.

### Assessments and outcomes

MSI status, tumour mutational burden (TMB), and programmed death-ligand 1 (PD-L1) expression were determined at the designated central laboratory; MSI status could also be analysed at study sites. Assessment of mismatch repair (MMR) was performed at study sites.

Tumour assessments were performed at baseline, every 6 weeks until week 48, and every 12 weeks thereafter. The primary endpoint was ORR assessed by independent radiological review committee (IRRC) per RECIST v1.1; confirmation of complete response and partial response was required after 28 days. Secondary efficacy endpoints included ORR assessed by IRRC (per iRECIST) and by investigators (per RECIST v1.1 and iRECIST), disease control rate (DCR; stable disease [SD] was determined ≥42 days from first study treatment), overall survival (OS), 6- and 12-month OS rate, progression-free survival (PFS), 6- and 12-month PFS rate, and duration of response (DOR). DCR, PFS, and DOR were assessed both by IRRC and by investigators per RECIST v1.1 and iRECIST.

Other secondary endpoints included safety, pharmacokinetics (PK), immunogenicity, and health-related quality of life (HRQoL). Safety was monitored throughout the trial and for 90 days after treatment discontinuation. AEs were coded according to Medical Dictionary for Regulatory Activities (MedDRA) v23.1 and graded per National Cancer Institute Common Terminology Criteria for Adverse Events v5.0. Adverse events of special interest included infusion-related reactions and immune-related adverse events (irAEs) [21]. Serplulimab serum concentrations were determined for PK assessment. Immunogenicity was assessed by antidrug antibodies (ADAs) and neutralising antibodies (NAbs) against serplulimab, and patients were considered ADA or NAb positive if they had at least one positive ADA or NAb result. HRQoL was evaluated by quality of life questionnaires. Additional assessment methods are provided in Supplementary Material.

### Statistical analysis

Assuming an ORR of 30%, a sample size of 40 patients were required to demonstrate that the lower limit of the 95% confidence interval (95% CI) for ORR was no lower than 15% at a one-sided 2.5% *α*-level. To account for censoring, dropout, and false positivity in detecting MSI-H/dMMR, the plan was to enrol around 100 patients.

Primary and secondary efficacy endpoints were analysed primarily in the main efficacy analysis population (MEAP). Subgroup analyses were conducted to investigate the impacts of PD-L1 expression (positive or negative), TMB status (low or high), MSI status (MSS/MSI-L or MSI-H), and tumour types (CRC or non-CRC) on efficacy. The efficacy endpoints were also analysed in the special-interest efficacy analysis population (SIEAP) and the sensitivity analysis population (SAP), two subsets of MEAP. Point estimates and Clopper–Pearson 95% CIs were calculated for ORR and DCR. OS, PFS, and DOR were estimated using the Kaplan–Meier method. The threshold for statistical success was a lower limit of the 95% CI of the primary endpoint being ≥15% in both the MEAP and the SIEAP. Safety was assessed in the safety set (SS) and in the MEAP. PK was analysed in the pharmacokinetic set. Immunogenicity assessment was based on the SS. Safety, HRQoL, PK, and immunogenicity data were summarised by descriptive statistics. Detailed definitions of the analysis sets are provided in Supplementary Material.

Statistical analysis was conducted using the SAS software, v9.4 or above (SAS Institute, NC, USA).

### Reporting summary

Further information on research design is available in the Nature Research Reporting Summary linked to this article.

## Results

### Patient characteristics and disposition

**Fig. 1:**
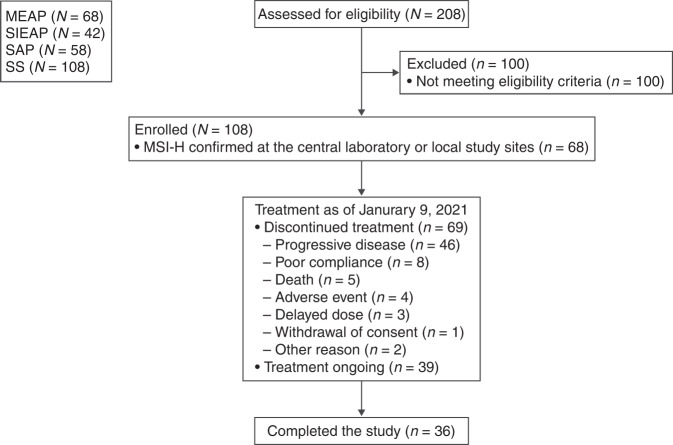
Participant flow diagram. MEAP main efficacy analysis population, MSI-H microsatellite instability-high, SAP sensitivity analysis population, SIEAP special-interest efficacy analysis population, SS safety set.

Between 22 July 2019 and 9 January 2021, 208 patients were screened, and 108 patients were enrolled (Fig. 1). The main reason for exclusion was not meeting the eligibility criteria. A total of 108 patients received at least one dose of serplulimab and comprised the SS. Of these patients, 68 had MSI-H tumours and were included in the MEAP; 58 and 42 were included in the SAP and the SIEAP, respectively.

In the MEAP, CRC was the most common tumour type (53 [77.9%]). A total of 30 (44.1%) patients were PD-L1 positive, and 55 (80.9%) were TMB-high, with a median TMB of 33.0 (Table 1). Baseline characteristics of patients in the SIEAP and SS were largely similar to those in the MEAP, except for a higher proportion of patients with TMB-H tumours in the MEAP (80.9%) and SIEAP (71.4%) than the SS (52.8%).

**Table 1:** Baseline characteristics.

Characteristic	Main efficacy analysis population (*n* = 68)	Special-interest efficacy analysis population (*n* = 42)	Safety set (*n* = 108)
Median age (range), years	53.0 (23.0–72.0)	53.5 (28.0–68.0)	55.0 (23.0–74.0)
Male, *n* (%)	36 (52.9)	19 (45.2)	55 (50.9)
Han Chinese, *n* (%)	68 (100)	42 (100)	108 (100)
Eastern Cooperative Oncology Group performance status, *n* (%)			
0	25 (36.8)	15 (35.7)	41 (38.0)
1	43 (63.2)	27 (64.3)	67 (62.0)
Stage M1, *n* (%)	67 (98.5)	42 (100)	106 (98.1)
Prior lines of therapy (chemotherapy), *n* (%)			
1	27 (39.7)	6 (14.3)	30 (27.8)
2	16 (23.5)	13 (31.0)	29 (26.9)
≥3	25 (36.8)	23 (54.8)	49 (45.4)
Primary tumour type, *n* (%)			
Colorectal cancer	53 (77.9)	28 (66.7)	73 (67.6)
Endometrial cancer	5 (7.4)	5 (11.9)	8 (7.4)
Gastric cancer	4 (5.9)	3 (7.1)	8 (7.4)
Other	6 (8.8)	6 (14.3)	19 (17.6)
Prior antitumour chemotherapy, *n* (%)			
Any	68 (100)	42 (100)	108 (100)
Oxaliplatin	57 (83.8)	34 (81.0)	81 (75.0)
Capecitabine	48 (70.6)	31 (73.8)	73 (67.6)
Fluorouracil	37 (54.4)	27 (64.3)	54 (50.0)
Irinotecan	34 (50.0)	31 (73.8)	52 (48.1)
Docetaxel	7 (10.3)	6 (14.3)	11 (10.2)
Other prior antitumour systemic therapy, *n* (%)			
Any	41 (60.3)	28 (66.7)	65 (60.2)
Bevacizumab	28 (41.2)	20 (47.6)	43 (39.8)
Cetuximab	7 (10.3)	7 (16.7)	12 (11.1)
PD-L1^a^			
Positive	30 (44.1)	17 (40.5)	43 (39.8)
Negative	29 (42.6)	21 (50.0)	54 (50.0)
Missing	9 (13.2)	4 (9.5)	11 (10.2)
Tumour mutational burden^b^			
High	55 (80.9)	30 (71.4)	57 (52.8)
Low	8 (11.8)	7 (16.7)	41 (38.0)
Missing	5 (7.4)	5 (11.9)	10 (9.3)

*PD-L1* programmed death-ligand 1.

^a^Positive PD-L1 status was defined as a combined positive score ≥1.

^b^Tumour mutational burden (TMB) high was defined as a score ≥10.

As of data cutoff (9 January 2021), the median duration of follow-up in the MEAP was 7.7 months (range, 1.1–16.4). The median duration of treatment exposure was 210 days; 11 patients completed the study, and 31 discontinued treatment due to PD (19 [27.9%]), poor compliance (4 [5.9%]), death (3 [4.4%]), AE (2 [2.9%]), delayed dose (2 [2.9%]), and other reason (1 [1.5%]). The numbers of patients who completed the study and those who discontinued treatment were 36 and 69 in the SS and 7 and 23 in the SIEAP, respectively.

### Efficacy

**Fig. 2:**
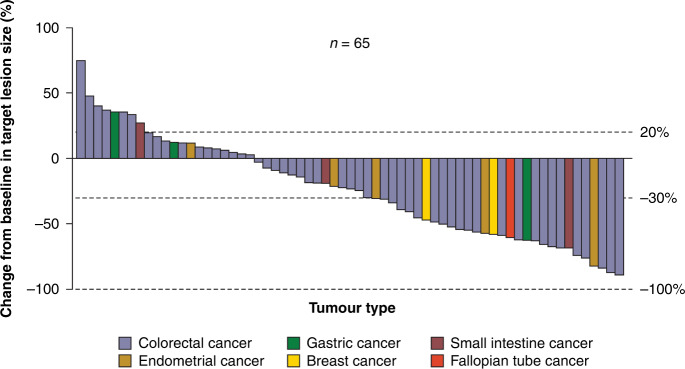
Best percentage change from baseline in target lesion size assessed by IRRC per RECIST v1.1. Analysis was based on the main efficacy analysis population. Three patients did not have tumour assessments and were therefore excluded. IRRC independent radiological review committee, RECIST Response Evaluation Criteria in Solid Tumors.

In the MEAP, a confirmed objective response by IRRC per RECIST v1.1 was observed in 26 patients (ORR, 38.2%; 95% CI, 26.7–50.8), including 2 (2.9%) patients with complete response and 24 (35.3%) with partial response (Table 2), with the lower limit of 95% CI of ORR meeting the prespecified threshold of positive results (≥15%). In addition, 20 (29.4%) patients had SD by IRRC per RECIST v1.1, contributing to a DCR of 67.6% (95% CI, 55.2–78.5). The best percentage change from baseline in target lesion size in the MEAP is presented in Fig. 2. The median DOR was not reached, with 95.7% (95% CI, 72.9–99.4) of patients estimated to have a DOR of ≥6 and ≥12 months. Similarly, in the SIEAP, IRRC-assessed ORR per RECIST v1.1 was 31.0% (95% CI, 17.6–47.1), which also met the threshold for statistical success (Table 2). IRRC-assessed DCR per RECIST v1.1 was 54.8% (95% CI, 38.7–70.2). Median DOR was not reached in this population, with 90.9% (95% CI, 50.8–98.7) of patients estimated to have a DOR of ≥6 and ≥12 months.

**Table 2:** Tumour response assessed by IRRC per RECIST v1.1.

Response	Main efficacy analysis population (*n* = 68)	Special-interest efficacy analysis population (*n* = 42)
Objective response rate		
* n* (%)	26 (38.2)	13 (31.0)
95% CI	26.7–50.8	17.6–47.1
Disease control rate		
* n* (%)	46 (67.6)	23 (54.8)
95% CI	55.2–78.5	38.7–70.2
Complete response, *n* (%)	2 (2.9)	1 (2.4)
Partial response, *n* (%)	24 (35.3)	12 (28.6)
Stable disease, *n* (%)	20 (29.4)	10 (23.8)
Progressive disease, *n* (%)	18 (26.5)	16 (38.1)
Non-evaluable^a^, *n* (%)	4 (5.9)	3 (7.1)
Median duration of response (95% CI), months	NR (NR–NR)	NR (NR–NR)
Response duration ≥6 months, % (95% CI)	95.7 (72.9–99.4)	90.9 (50.8–98.7)
Response duration ≥12 months, % (95% CI)	95.7 (72.9–99.4)	90.9 (50.8–98.7)

*CI* confidence interval, *IRRC* independent radiological review committee, *NR* not reached, *RECIST* Response Evaluation Criteria in Solid Tumors.

^a^Three and two patients did not have tumour assessments in the main efficacy analysis population and the special-interest efficacy analysis population, respectively. One patient included in both populations had stable disease as the best response, but the time from first study treatment to tumour assessment date was 38 days, which was shorter than the protocol specified minimum time from baseline (≥42 days), and thus tumour response was downgraded to non-evaluable.

**Fig. 3:**
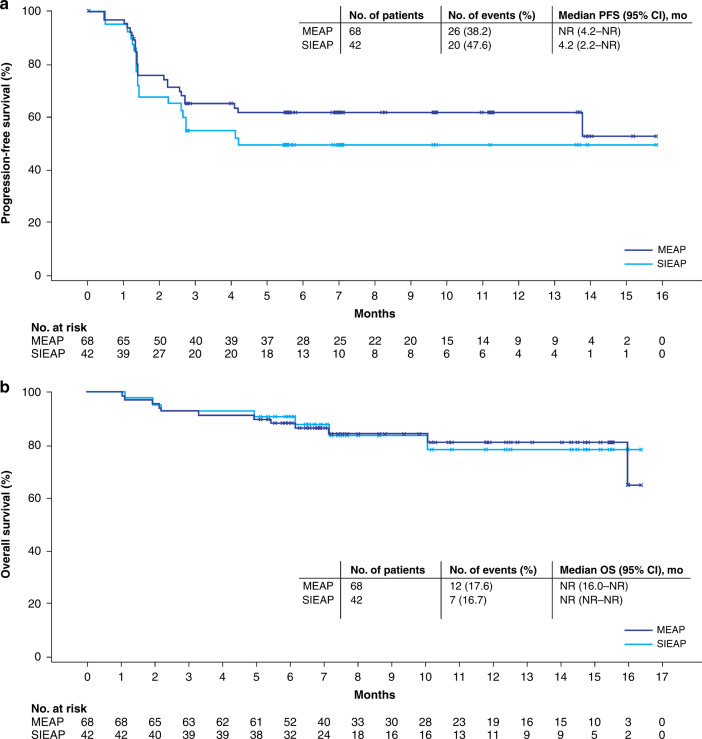
Survival in the MEAP and SIEAP. Kaplan–Meier curves of progression-free survival (**a**) and overall survival (**b**). Tumour response was assessed by IRRC per RECIST v1.1. CI confidence interval, IRRC independent radiological review committee, MEAP main efficacy analysis population, mo month, NR not reached, OS overall survival, PFS progression-free survival, RECIST Response Evaluation Criteria in Solid Tumors, SIEAP special-interest efficacy analysis population.

Twenty-six (38.2%) patients in the MEAP had PFS events (23 had PD as assessed by IRRC per RECIST v1.1, and 3 died without PD). Median PFS was not reached (Fig. 3). The estimated 6- and 12-month PFS rates were both 61.9% (95% CI, 49.0–72.5). In the SIEAP, median PFS (by IRRC per RECIST v1.1) was 4.2 months (95% CI, 2.2–not reached [NR]), with estimated 6- and 12-month PFS rates being 49.7% (95% CI, 33.4–64.1).

A consistently favourable antitumour activity for serplulimab was demonstrated by IRRC assessments per iRECIST and by investigator assessments per RECIST v1.1 or iRECIST (Supplementary Tables S1 and S2).

Twelve patients in the MEAP had died by data cutoff (Fig. 3). Median OS was not reached (95% CI, 16.0–NR). The estimated 6- and 12-month OS rates were 88.2% (95% CI, 77.7–93.9) and 81.2% (95% CI, 67.8–89.4), respectively. OS was similar in the SIEAP (median OS [95% CI], NR [NR–NR]; 6-month OS rate, 90.5% [76.6–96.3]; 12-month OS rate, 78.4% [58.3–89.6]). Efficacy results were consistent in the SAP (Supplementary Table S3).

Subgroup analysis was based on tumour assessments by IRRC per RECIST v1.1 and OS. Among all enrolled patients, MSI-H patients achieved a greater ORR (38.2% vs 2.8%), a greater DCR (67.6% vs 19.4%), a longer PFS (median PFS [95% CI], NR [4.2–NR] vs 1.4 months [1.3–1.6]), and a longer OS (median OS, NR [16.0–NR] vs 5.0 months [4.0–9.4]) compared with MSS/MSI-L patients. In the MEAP, ORR was numerically higher in PD-L1-positive patients (Supplementary Table S4). Median PFS and median OS were longer in TMB-high patients, while the differences in ORR and DCR were not pronounced. ORR was similar between patients with CRC and those with non-CRC tumours, although there was a trend of longer DOR in CRC patients.

Among patients in the MEAP who had discontinued or completed serplulimab treatment, 8 (11.8%) patients received antitumour chemotherapy, 3 (4.4%) received subsequent radiation therapy, 2 (2.9%) received surgery, and 15 (22.1%) were treated with other antitumour therapies.

### Quality of life

Most patients experienced improved or stable HRQoL relative to baseline as assessed by quality of life questionnaires (Supplementary Tables S5 and S6).

### Safety

Among patients in the SS, 105 (97.2%) patients reported at least one treatment-emergent adverse event (TEAE) (Table 3). The most common TEAEs were anaemia (34.3%), hypoproteinaemia (27.8%), and increased aspartate aminotransferase (25.0%). Grade ≥3 TEAEs occurred in 53 (49.1%) patients, the most common being anaemia (8.3%), PD (6.5%), increased gamma-glutamyltransferase (5.6%), and intestinal obstruction (5.6%). Adverse drug reactions (ADRs) occurred in 86 (79.6%) patients (Table 3 and Supplementary Table S7), and most events were grade 1 or 2. Serious ADRs occurred in 16 (14.8%) patients. Three (2.8%) patients discontinued serplulimab treatment because of ADRs, including abnormal liver function (2 [1.9%]), immune-mediated liver injury (1 [0.9%]), pneumonitis (1 [0.9%]), and fever (1 [0.9%]). Three (2.8%) patients had grade 5 ADRs as assessed by investigators: one had intestinal obstruction and one experienced PD after receiving serplulimab, and both events were considered as possibly related to the study drug; one patient experienced PD after withdrawal, with the exact cause of death unknown.

**Table 3:** Treatment-emergent adverse events.

Adverse events	Safety set (*n* = 108)	Main efficacy analysis population (*n* = 68)
Any TEAEs	105 (97.2)	67 (98.5)
Grade ≥3	53 (49.1)	28 (41.2)
Grade 5	15 (13.9)	5 (7.4)
TEAEs leading to treatment discontinuation	4 (3.7)	2 (2.9)
Serious TEAEs	32 (29.6)	16 (23.5)
irAEs	52 (48.1)	39 (57.4)
Any ADRs	86 (79.6)	58 (85.3)
Grade ≥3	27 (25.0)	18 (26.5)
Grade 5	3 (2.8)	2 (2.9)
ADRs leading to treatment discontinuation	3 (2.8)	2 (2.9)
Serious ADRs	16 (14.8)	10 (14.7)
TEAEs of grade ≥3 in ≥5% of patients in the SS		
Anaemia	9 (8.3)	4 (5.9)
Disease progression	7 (6.5)	2 (2.9)
Gamma-glutamyltransferase increased	6 (5.6)	3 (4.4)
Intestinal obstruction	6 (5.6)	5 (7.4)
irAEs in ≥3% patients in the SS		
Hypothyroidism	18 (16.7)	13 (19.1)
Hyperthyroidism	9 (8.3)	7 (10.3)
Pneumonitis	5 (4.6)	1 (1.5)
Thyroid-stimulating hormone increased	4 (3.7)	2 (2.9)
Abnormal liver function	4 (3.7)	4 (5.9)

*ADR* adverse drug reaction, *irAE* immune-related adverse event, *MSI-H* microsatellite instability-high, *SS* safety set, *TEAE* treatment-emergent adverse event.

Adverse events of special interest occurred in 52 (48.1%) patients and all were irAEs, the most common being hypothyroidism (18 [16.7%]) and hyperthyroidism (9 [8.3%]; Table 3). Most irAEs were grade 1 or 2 in severity, and grade ≥3 irAEs occurred in 10 (9.3%) patients; there were no grade 5 irAEs. No infusion-related reactions occurred in this study.

The incidences and severity of TEAEs in the MEAP were largely consistent with those in the SS (Table 3).

### Pharmacokinetics

The mean trough concentration of serplulimab increased as treatment cycle increased, indicating an accumulation of serplulimab (Supplementary Fig. S1). There was a trend toward lower accumulation in terms of trough concentration in ADA-positive, PD-L1-negative, TMB-low, and MSS/MSI-L subgroups (Supplementary Table S8).

### Immunogenicity

ADAs were detected in 5/108 patients (4.6%). No patients had detectable NAbs.

## Discussion

Serplulimab treatment resulted in durable and clinically meaningful tumour responses in Chinese patients with previously treated unresectable or metastatic MSI-H solid tumours. This study met the prespecified primary endpoint. ORR (38.2% as assessed by IRRC per RECIST v1.1) was comparable with that observed with pembrolizumab in MSI-H/dMMR solid tumours (39.6% based on a pooled analysis of five trials [14]) and with that for nivolumab in MSI-H/dMMR CRC (31.1%) [22]. Most of the patients still had ongoing tumour response at the data cutoff, with an estimated 95.7% of patients having a DOR of ≥1 year. The safety profile of serplulimab was consistent with that for PD-1 inhibitors.

Serplulimab demonstrated a sustained effect on MSI-H solid tumours, and this benefit was observed irrespective of previous lines of therapy. Patients with CRC and those with non-CRC tumours seemed to have similar responses to serplulimab treatment based on the ORR, though further studies with large sample sizes are needed to confirm the result. Moreover, the durable response was coupled with maintenance or improvements of HRQoL in most patients. The mature data on PFS and OS from this ongoing trial will provide further insight into the clinical efficacy.

Tumour responses assessed by RECIST v1.1 and iRECIST in this study provided additional evidence for the antitumour activity of serplulimab. In this study, outcomes were mostly consistent when evaluated by RECIST and by iRECIST. However, median PFS was shorter in the SIEAP when assessed per RECIST v1.1 than according to iRECIST (4.2 vs NR by IRRC or by the investigators), which may be due to misclassification of pseudoprogression as PD by RECIST v1.1. Nevertheless, similar outcomes according to assessment by IRRC and by the investigators add to the robustness of the tumour response results.

Discordance between MMR protein testing and MSI DNA testing results have been reported by previous studies, ranging from 1 to 10% [23, 24]. In our study, MSI-H and dMMR overlapped partially, as 68 patients were MSI-H out of 108 patients identified as MSI-H or dMMR. The discordance may be due to differences in the sensitivity and specificity of techniques used, misinterpretation of testing results, or possibly biological reasons, such as functional redundancy of proteins for DNA mismatch repair and MSI-H originating from other genetic defects [24–27]. Caution should be taken when using MMR immunohistochemistry testing as a screening tool given occasional equivocal staining patterns, sometimes inadequate sensitivity due to antibodies used, requirements of pathologist’s experience, and other pitfalls [23–26]. To circumvent these pitfalls, we used MSI DNA testing to select target patient population for serplulimab; MSI-H as a predictor of tumour response to serplulimab was supported by a better response in MSI-H patients than MSS/MSI-L patients.

Understanding the response to immune checkpoint inhibitors in MSI-H tumours is of interest, with high TMB shown to be predictive of better clinical outcomes in MSI-H cancers treated with immune checkpoint inhibitors [28, 29]. In contrast, tumour response to PD-1 inhibitors was found to be consistent across PD-L1 positive and negative MSI-H/dMMR tumours in the KEYNOTE-016 and CheckMate 142 studies [22, 30]. Although numerical differences were observed in terms of ORR or PFS in our subgroup analyses according to PD-L1 expression and TMB, results were inconclusive given the small differences between groups and the small sample size, especially in the TMB-low group.

The TEAEs reported in this study were in line with AE profiles for other immunotherapies [14, 31]. The most frequent grade ≥3 events and irAEs are also commonly observed during treatment with pembrolizumab in solid tumour patients [32, 33]. For the three patients with grade 5 ADRs as determined by investigators, the sponsor assessment suggested that these cases were possibly unrelated to serplulimab, but probably due to PD as well as peritoneal metastasis and adhesion (for the case with intestinal obstruction), and underlying diseases (for two cases with PD).

Limitations of this study included lack of a comparator, a small sample size, and overrepresentation of CRC patients. Moreover, the interpretation of subgroup analysis by TMB was limited by the small number of patients who were TMB-low. As *RAS* mutation was shown to be associated with a shorter PFS in MSI-H/dMMR CRC patients treated with pembrolizumab in first-line setting [34], subgroup analysis according to *BRAF*, *KRAS*, and *NRAS* mutation status is recommended in future studies.

In conclusion, serplulimab provided encouraging efficacy and manageable safety profile in Chinese patients with unresectable or metastatic MSI-H solid tumours who have progressed on or been intolerant to at least one prior line of standard therapy, regardless of tumour type. Based on these results, the China National Medical Products Administration has granted priority review designation to serplulimab for treating this patient population.

## Supplementary information

Supplementary Material

Reporting guidelines-CONSORT Checklist

Reporting Summary (reproducibility checklist)

## Data Availability

The data generated in this study are available from the corresponding author upon reasonable request.
