# Correction To: Serplulimab, a novel anti-PD-1 antibody, in patients with microsatellite instability-high solid tumours: an open-label, single-arm, multicentre, phase II trial

**DOI:** 10.1038/s41416-022-02043-7

**Published:** 2022-11-02

**Authors:** Shukui Qin, Jin Li, Haijun Zhong, Chuan Jin, Lili Chen, Xianglin Yuan, Qingxia Fan, Kehe Chen, Peiguo Cao, Jianjun Xiao, Da Jiang, Tao Zhang, Hongyu Zhang, Xicheng Wang, Wei Wang, Lin Han, Qingyu Wang, Jun Zhu, the Serplulimab-MSI-H Investigators

**Affiliations:** 1 Department of Oncology, Qinhuai Medical Area, Eastern Theater General Hospital of PLA China, Nanjing, China; 2 Department of Oncology, Tongji University Shanghai East Hospital, Shanghai, China; 3 Department of Abdominal Oncology, Cancer Hospital of the University of Chinese Academy of Sciences (Zhejiang Cancer Hospital), Hangzhou, China; 4 Department of Oncology, Affiliated Cancer Hospital and Institute of Guangzhou Medical University, Guangzhou, China; 5 Department of Hematology and Oncology, Taizhou First People’s Hospital, Taizhou, China; 6 Department of Oncology, Tongji Hospital, Huazhong University of Science and Technology, Wuhan, China; 7 Department of Oncology, The First Affiliated Hospital of Zhengzhou University, Zhengzhou, China; 8 Department of Oncology, The People’s Hospital of Guangxi Zhuang Autonomous Region, Nanning, China; 9 Department of Oncology, The Third Xiangya Hospital of Central South University, Changsha, China; 10 Chemotherapeutic Department, Zhongshan City People’s Hospital, Zhongshan, China; 11 Department of Oncology, The Fourth Hospital of Hebei Medical University, Shijiazhuang, China; 12 Department of Oncology, The First Affiliated Hospital of Chongqing Medical University, Chongqing, China; 13 Cancer Center, The Fifth Affiliated Hospital Sun Yat-sen University, Zhuhai, China; 14 Department of Oncology, The First Affiliated Hospital of Guangdong Pharmaceutical University, Guangzhou, China; 15 Department of Gastrointestinal Oncology, The First People’s Hospital of Foshan, Foshan, China; 16 Shanghai Henlius Biotech, Inc., Shanghai, China

**Keywords:** Cancer therapy, Biomarkers, Phase II trials, Immunotherapy

Correction to: *British Journal of Cancer* 10.1038/s41416-022-02001-3, published online 19 October 2022

The original version of this article contained a mistake in the PDF as the corresponding author Jin Li is missing from the Consortia information. We apologize for the error. The original article has been corrected.

