# Supplementary material for: Serplulimab, a novel anti-PD-1 antibody, in patients with microsatellite instability-high solid tumours: an open-label, single-arm, multicentre, phase II trial
Source: Br J Cancer. 2022 Oct 19;127(12):2241–8. doi: 10.1038/s41416-022-02001-3 (PMC9726893; doi:10.1038/s41416-022-02001-3)
Supplement: Supplementary file 1 — Supplementary Material [file 41416_2022_2001_MOESM1_ESM.pdf]

SUPPLEMENTARY MATERIAL

Inclusion criteria

Subjects must have met all following inclusion criteria to be enrolled in the study:

1. Volunteer to participate in this clinical study; fully understand the study and be willing to sign the informed consent form (ICF); be willing to follow and be able to complete all study procedures.
2. Age ≥18 years and ≤75 years when signing the ICF.
3. Subjects with unresectable or metastatic microsatellite instability-high (MSI-H) or mismatch repair-deficient (dMMR) malignant solid tumours which are histologically and/or cytologically confirmed by the central laboratory or study sites.
4. Subjects who have progressed on or are intolerant to at least one prior line of current standard anticancer treatment, or refuse to receive subsequent treatment.
   Note: For radical concurrent chemoradiotherapy and neoadjuvant/adjuvant therapy (chemotherapy or chemoradiotherapy), if disease progression (PD) occurs during treatment or within 6 months after treatment discontinuation, it should be considered as first-line treatment failure; if PD occurs 6 months after treatment discontinuation, it does not count as first-line treatment failure. For first-line chemotherapy, dose should be reduced when subjects develop drug intolerance for the first time; if a patient remains intolerant to the drug when dose is reduced to 50% of the standard dose, then this patient is intolerant to chemotherapy. The standard first-line treatment for colorectal cancer refers to fluorouracil-based chemotherapy.
5. The interval between the end of previous systemic antitumour treatment and the first dose of this study must be ≥2 weeks. In addition, treatment-related adverse events must resolved to National Cancer Institute Common Terminology Criteria for Adverse Events (NCI CTCAE) v5.0 grade ≤1 (excluding grade 2 alopecia). The interval between the end of previous treatment with traditional Chinese medicine, proprietary Chinese medicine, and

immunomodulators (e.g., thymosin, lentinan, and interleukin-12) and the first dose of this study must be ≥2 weeks.

6. Subjects must have at least one measurable lesion as assessed by independent radiological review committee (IRRC) per Response Evaluation Criteria in Solid Tumors (RECIST) v1.1. Note: Measurable lesions must be situated in a previously non-irradiated area. If the lesion in the previously irradiated area is the only available target lesion, the investigator should provide imaging data of the lesion before and after significant progression.
7. Subjects must provide tumour tissues and blood samples for the assessment of microsatellite instability (MSI), tumour mutational burden (TMB), and programmed death-ligand 1 (PD-L1) expression level. Subjects with available test results of all these biomarkers from the central laboratories appointed by this study can be exempt from retesting.
   Note: Formalin-fixed tumour specimens collected from non-irradiated area within 6 months prior to the first dose of the investigational product are recommend. Paraffin-embedded tumour specimens (preferred), archival formalin-fixed and paraffin-embedded (FFPE) tumour specimens, and unstained fresh serial tissue sections (on slide) are acceptable. Relevant pathological reports of the tumour specimens are also required. Freshly collected specimens, excision, core needle biopsy, resection, incision, puncture, or forceps biopsies are within the acceptable range (fresh tissues preferred). Needle aspiration specimens (i.e., specimens lacking intact tissue structure), brush specimens, or cell precipitates from pleural or peritoneal effusions are not acceptable. Refer to the laboratory manual for detailed specimen requirements.
8. Eastern Cooperative Oncology Group (ECOG) performance status score should be 0 or 1 within 7 days before the first dose of the investigational product.
9. Life expectancy ≥12 weeks.
10. Subjects should be hepatitis B surface antigen (HBsAg) negative. Subjects with positive HBsAg and/or positive hepatitis B core antibody (HBcAb) are eligible only if their hepatitis B virus DNA (HBV-DNA) levels are <2000 IU/mL or <10^4 copies/mL. If higher than this limit,

subjects should first receive antiviral treatment; their HBV-DNA levels need to be within the normal range for at least two weeks, and they must be on antiviral treatment throughout the study. For subjects receiving antiviral treatment during screening, they should also be on antiviral treatment throughout the study.

11. Subjects should have adequate major organ function and meet the following criteria (see table below). Subjects should not receive blood transfusion or treatment with albumin, recombinant human thrombopoietin or colony-stimulating factor within 14 days before the first dose of the investigational product.

**Complete blood count**	
Absolute neutrophil count (ANC)	$\geq 1.5 \times 10^9$/L
Platelets (PLTs)	$\geq 100 \times 10^9$/L
Haemoglobin (Hb)	≥90 g/L
**Liver function test**	
Total bilirubin (TBIL)	≤1.5 × upper limit of normal (ULN)
Alanine aminotransferase (ALT)	≤2.5 × ULN;<br>≤5 × ULN for subjects with liver metastases;<br>≤3 × ULN for subjects with positive anti-hepatitis C virus (HCV) antibody or positive HCV-RNA
Aspartate aminotransferase (AST)	≤2.5 × ULN;<br>≤5 × ULN for subjects with liver metastases;<br>≤3 × ULN for subjects with positive anti-HCV antibody or positive HCV-RNA
Albumin	≥30 g/L
**Kidney function test**	
Creatinine (Cr)	≤1.5 × ULN;<br>If >1.5 × ULN, creatinine clearance should be ≥50 mL/min (calculated by Cockcroft-Gault equation)
**Coagulation test**	
Activated partial thromboplastin time (aPTT)	≤1.5 × ULN
Prothrombin time (PT)	≤1.5 × ULN
International normalized ratio (INR)	≤1.5 × ULN
**Routine urine test/24-hour urine protein**	
Urine protein	Urine protein (qualitative) ≤1+;

	If ≥2+, 24-hour urine protein test is required, and if 24-hour urine protein is <1 g, enrolment is allowed

12. Female subjects must meet the following criteria:
    a. Menopause (defined as no menstruation for at least one year with no confirmed cause other than menopause); or
    b. Received surgical sterilization (ovariectomy and/or hysterectomy); or
    c. With childbearing potential but meet the following criteria:
        i. Serum pregnancy test must be negative within 7 days before the first dose; and
        ii. Agree to take effective contraceptive methods throughout the trial and for at least 120 days after the last dose of the investigational product; and
        iii. Agree not to breastfeed.
13. Male subjects must agree to take effective contraceptive methods throughout the trial and for at least 120 days after the last dose of the investigational product.

Exclusion criteria

Subjects meeting any of the following criteria will not be enrolled in the study:

1. Subjects who plan to undergo or previously underwent organ or bone marrow transplantation.
2. Pleural effusion, pericardial effusion or ascites that cannot be controlled with appropriate intervention.
3. Subjects with known or screening-detected active central nervous system (CNS) metastasis and/or carcinomatous meningitis. However, the following subjects are allowed to be enrolled:
    a. Subjects with asymptomatic brain metastasis (i.e., without progressive CNS symptoms caused by brain metastatic lesions, no requirement for corticosteroids, and lesion size ≤1.5 cm) are allowed to participate in this study, but regular brain imaging of disease sites is necessary.
    b. For subjects with brain metastases after treatment, they are eligible for inclusion if their brain metastatic lesions have been stable for at least 1 month, without evidence

of new or increased brain metastases, and with steroids discontinued 3 days prior to the first dose of the investigational product. Stable brain metastasis should be defined before the first dose of the investigational product.

4. Subjects with spinal cord compression which cannot be treated radically by surgery and/or radiotherapy, or subjects previously diagnosed with spinal cord compression with no post-treatment clinical evidence showing stable disease for ≥1 week before the first dose of the investigational product.
5. Imaging tests show clear signs of tumour invasion of thoracic great vessels.
6. Occurrence of cerebrovascular accident (excluding lacunar cerebral infarction, mild cerebral ischaemia, and transient ischemic attack), myocardial infarction, unstable angina pectoris, or poorly controlled arrhythmia (including QTc interval ≥450 ms for males and ≥470 ms for females; QTc interval is calculated by Fridericia formula) within six months before the first dose of the investigational product.
7. New York Heart Association (NYHA) class III or IV heart failure, or colour echocardiogram showing left ventricular ejection fraction (LVEF) <50%.
8. Presence of NCI CTCAE v5.0 grade ≥2 peripheral neuropathy.
9. Infection with human immunodeficiency virus (HIV).
10. HBsAg or HBcAb positive, and HCV antibody positive.
11. Presence of active pulmonary tuberculosis.
12. Previously or currently suffering from interstitial pneumonia, pneumoconiosis, radiation pneumonitis, drug-related pneumonitis, severely impaired lung function and other conditions which may interfere with the detection and treatment of suspected drug-related pulmonary toxicity.
13. Presence of known active or suspected autoimmune diseases. Subjects with stable disease and require no systemic immunosuppressive therapy are eligible for enrolment.
14. Received live vaccines within 28 days before the first dose of the investigational product.

15. Subjects requiring treatment with systemic corticosteroids (prednisone >10 mg/day or equivalent) or other immunosuppressive therapies within 14 days before the first dose of the investigational product or during the study period. However, subjects with no active autoimmune disease and require only inhaled or topical steroids, or adrenal hormone replacement therapy at a dose equivalent to prednisone ≤10 mg/day are allowed to be enrolled.
16. Presence of any active infection requiring systemic anti-infective treatment within 14 days before the first dose of the investigational product.
17. Subjects who have received major surgery within 28 days before the first dose of the investigational product. Major surgery is defined as surgeries requiring at least three weeks to recover before initiating treatment with the investigational product. Subjects receiving tumour puncture or lymph node biopsy are allowed to be enrolled.
18. Subjects who have received radical radiotherapy within 3 months before the first dose of the investigational product.

    Note: Palliative radiotherapy for bone or superficial lesions is allowed if it has been completed 14 days before the first dose (refer to the local standard of care for the course of treatment). Radiotherapy covering more than 30% of the bone marrow area within 28 days prior to the first dose is not allowed.
19. Subjects who may receive other antitumour treatments such as chemotherapy, targeted therapy or radiotherapy (excluding palliative radiotherapy) during the study period.
20. Subjects who have previously received any T cell co-stimulation or immune checkpoint therapies, including but not limited to cytotoxic T-lymphocyte-associated antigen 4 (CTLA-4) inhibitors, programmed death 1 (PD-1) inhibitors, programmed death-ligand 1/2 (PD-L1/2) inhibitors, and other drugs targeting T cells.
21. Subjects are under the treatment period of other clinical studies, or the time between the planned treatment initiation of this study and the end of the treatment period of the previous clinical study is less than 14 days.

22. Known history of severe allergy to any monoclonal antibody or excipients of the investigational product.
23. Pregnant or lactating women.
24. Known history of psychotropic substance abuse or drug addiction. Subjects who agree not to drink alcohol during the study period are allowed to be enrolled.
25. Subjects with other risk factors which may lead to premature termination of this study at the discretion of the investigators.

Additional assessment methods

Biomarkers

MSI status, PD-L1 expression, and TMB were analysed in archival FFPE tumour samples collected within 6 months before initiating study treatment, or if not available, in fresh biopsy samples collected during screening. MSI status and TMB were analysed using validated next-generation sequencing (NGS)-based methods developed by GeneCast Biotechnology (Jiangsu, China) and performed on Illumina NovaSeq platform (Illumina, CA, USA). MSI status was determined based on 24 mononucleotide loci including BAT-25, BAT-26, NR-21, NR24, and MONO-27; MSI-H was defined as ≥30% loci showing instability. Compared with the fluorescent PCR-based MSI assay developed by Promega (MSI Analysis System, Version 1.2), this NGS-based MSI assay had a sensitivity of 100%, a specificity of 98.2%, and an accuracy of 98.7%. TMB-high was defined as a TMB of ≥10 mutations per megabase. PD-L1 expression was analysed by MEDx Translational Medicine (Jiangsu, China) using an immunohistochemistry (IHC) assay kit with a monoclonal mouse antihuman PD-L1 antibody, clone 22C3 (Agilent, CA, USA). Combined positive score (CPS) was calculated, which is the number of PD-L1 staining cells (tumour cells, lymphocytes, macrophages) divided by the total number of viable tumour cells, multiplied by 100. Positive PD-L1 status was defined as a CPS ≥1. Assessment of mismatch repair (MMR) was performed on FFPE tumour samples or fresh biopsies by IHC of MMR proteins.

Efficacy

Tumour assessments with enhanced computed tomography or magnetic resonance imaging were performed at baseline, every 6 weeks until week 48, and every 12 weeks thereafter. Tumour response was assessed both by IRRC and by investigators per RECIST v1.1 and response criteria for cancer immunotherapy trials (iRECIST). Patients who discontinued treatment for reasons other than PD were followed until progression, initiation of a new antitumour therapy, withdrawal of consent, loss to follow-up, death, or study completion. Survival was assessed until loss to follow-up, death, or study completion, whichever occurred first.

Safety

Safety was monitored throughout the trial and for 90 days after treatment discontinuation by drug exposure, vital signs, physical examinations, laboratory tests, electrocardiogram, ECOG performance status, as well as incidence and severity of adverse events (AEs). AEs were coded according to Medical Dictionary for Regulatory Activities (MedDRA) v23.1 and graded per NCI CTCAE v5.0. Adverse events of special interest of serplulimab included infusion-related reactions and immune-related adverse events.

Quality of life

Patient's health-related quality of life was evaluated using the five-level version of the EuroQol five-dimensional questionnaire (EQ-5D-5L) and the European Organisation for Research and Treatment of Cancer Quality of Life Questionnaire-Core 30 (EORTC QLQ-C30) before initiating study treatment and alternate treatment cycles prior to treatment administration.

Pharmacokinetics

Blood samples for pharmacokinetics (PK) and immunogenicity assessments were collected prior to dosing in cycles 1, 2, 4, 6, 8, and every 4 cycles thereafter, as well as at treatment termination and 30 days after the last treatment; in addition, PK samples were also collected within two hours after dosing in cycles 1 and 8. Serplulimab serum concentrations were determined using a validated enzyme-linked immunosorbent assay (WuXi AppTec, Shanghai, China). The working range of this assay was 100–4000 ng/mL.

Immunogenicity

Immunogenicity was assessed by antidrug antibodies (ADAs) and neutralizing antibodies (NAbs) against serplulimab using Meso Scale Discovery electrochemiluminescent immunoassays (WuXi AppTec, Shanghai, China). For detection of ADAs, the screening sensitivity was 1.16 ng/mL, with a confirmatory sensitivity of 2.34 ng/mL; for detection of NAbs, the sensitivity was 0.17 μg/mL. Patients were considered ADA or NAb positive if they had at least one positive ADA or NAb result.

Statistical analysis sets

- Main efficacy analysis population (MEAP) included patients with MSI-H solid tumours detected at local sites or confirmed at the central laboratory who received at least one dose of serplulimab.
- Special-interest efficacy analysis population (SIEAP), a subset of MEAP, comprised patients with colorectal cancer who had received fluoropyrimidine, irinotecan, and oxaliplatin, those patients with gastric cancer who had received at least two lines of therapy, as well as patients with other tumour types who had received at least one line of therapy.
- Safety set (SS) included patients who received at least one dose of serplulimab and had at least one safety assessment post-baseline.
- Sensitivity analysis population (SAP) included patients with MSI-H solid tumours confirmed at the central laboratory who received at least one dose of serplulimab and had no major protocol deviations.
- Pharmacokinetic set (PKS) included patients who received at least one dose of serplulimab and provided at least one postdosing PK sample as planned without major protocol deviations that might impact PK assessment.

The Serplulimab-MSI-H Investigators and affiliations

Investigator name.... Affiliation

Shukui Qin .... Qinhuai Medical Area, Eastern Theater General Hospital of PLA China

Jin Li .... Tongji University Shanghai East Hospital

Haijun Zhong .... Cancer Hospital of the University of Chinese Academy of Sciences (Zhejiang Cancer Hospital)

Chuan Jin .... Affiliated Cancer Hospital and Institute of Guangzhou Medical University

Lili Chen .... Taizhou First People's Hospital

Xianglin Yuan .... Tongji Hospital, Huazhong University of Science and Technology

Qingxia Fan............... The First Affiliated Hospital of Zhengzhou University

Kehe Chen................. The People's Hospital of Guangxi Zhuang Autonomous Region

Peiguo Cao .... The Third Xiangya Hospital of Central South University

Jianjun Xiao .... Zhongshan City People's Hospital

Da Jiang .... The Fourth Hospital of Hebei Medical University

Tao Zhang .... The First Affiliated Hospital of Chongqing Medical University

Hongyu Zhang .... The Fifth Affiliated Hospital Sun Yat-sen University

Xicheng Wang .... The First Affiliated Hospital of Guangdong Pharmaceutical University

Wei Wang .... The First People's Hospital of Foshan

Qing Zhu .... West China Hospital of Sichuan University

Yunfeng Li .... Yunnan Cancer Hospital

Yuxian Bai .... Harbin Medical University Cancer Hospital

Zhendong Chen......... The Second Hospital of Anhui Medical University

Zhong Xie .... Affiliated Hospital of Guangdong Medical University

Changzheng Li.......... Shandong Cancer Hospital & Institute

Yongdong Jin............ Sichuan Cancer Hospital & Institute

Huangyang Ye .... The First Affiliated Hospital of Xiamen University

Fuxiang Zhou............ Zhongnan Hospital of Wuhan University

Xiubao Ren .... Tianjin Medical University Cancer Institute & Hospital

Xianli Yin .... Hunan Cancer Hospital, The Affiliated Cancer Hospital of Xiangya School of Medicine, Central South University

Shirong Cai .... The First Affiliated Hospital of Sun Yat-sen University

Jingdong Zhang......... Liaoning Cancer Hospital & Institute, Cancer Hospital of China Medical University

Yanhong Deng .... The Sixth Affiliated Hospital, Sun Yat-sen University

Yang Zhang............... The Second Hospital of Dalian Medical University

Jun Liang................... Peking University International Hospital

Xi Chen .... The 900th Hospital of Joint Logistic Support Force

Xiuwen Wang .... Qilu Hospital of Shandong University

Supplementary Table S1. Best overall response, duration of response, and progression-free survival by IRRC per iRECIST

	Main efficacy analysis population (*n* = 68)	Special-interest efficacy analysis population (*n* = 42)
Objective response rate		
n (%)	26 (38.2)	13 (31.0)
95% CI	26.7–50.8	17.6–47.1
Disease control rate		
n (%)	46 (67.6)	23 (54.8)
95% CI	55.2–78.5	38.7–70.2
Complete response, *n* (%)	2 (2.9)	1 (2.4)
Partial response, *n* (%)	24 (35.3)	12 (28.6)
Stable disease, *n* (%)	20 (29.4)	10 (23.8)
Unconfirmed progressive disease, *n* (%)	8 (11.8)	7 (16.7)
Confirmed progressive disease, *n* (%)	10 (14.7)	9 (21.4)
Non-evaluable, *n* (%)	4 (5.9)	3 (7.1)
Median duration of response (95% CI), months	NR (NR–NR)	NR (NR–NR)
Response duration ≥6 months, % (95% CI)	95.7 (72.9–99.4)	90.9 (50.8–98.7)
Response duration ≥12 months, % (95% CI)	95.7 (72.9–99.4)	90.9 (50.8–98.7)
Median progression-free survival (95% CI), months	NR (4.2–NR)	NR (2.2–NR)
6-month progression-free survival rate, % (95% CI)	62.9 (49.9–73.4)	51.0 (34.4–65.4)
12-month progression-free survival rate, % (95% CI)	62.9 (49.9–73.4)	51.0 (34.4– 65.4)

CI, confidence interval; iRECIST, response criteria for cancer immunotherapy trials; IRRC, independent radiological review committee; NR, not reached; RECIST, Response Evaluation Criteria in Solid Tumors.

Supplementary Table S2. Best overall response, duration of response, and progression-free survival by the investigators per RECIST version 1.1 or per iRECIST

	RECIST version 1.1		iRECIST	
	Main efficacy analysis population (*n* = 68)	Special-interest efficacy analysis population (*n* = 42)	Main efficacy analysis population (*n* = 68)	Special-interest efficacy analysis population (*n* = 42)
Objective response rate				
n (%)	24 (35.3)	12 (28.6)	25 (36.8)	13 (31.0)
95% CI	24.1–47.8	15.7–44.6	25.4–49.3	17.6–47.1
Disease control rate				
n (%)	47 (69.1)	24 (57.1)	48 (70.6)	25 (59.5)
95% CI	56.7–79.8	41.0–72.3	58.3–81.0	43.3–74.4
Complete response, *n* (%)	0	0	0	0
Partial response, *n* (%)	24 (35.3)	12 (28.6)	25 (36.8)	13 (31.0)
Stable disease, *n* (%)	23 (33.8)	12 (28.6)	23 (33.8)	12 (28.6)
Confirmed progressive disease, *n* (%)	17 (25.0)	15 (35.7)	7 (10.3)	7 (16.7)
Unconfirmed progressive disease, *n* (%)	NA	NA	9 (13.2)	7 (16.7)
Non-evaluable, *n* (%)	4 (5.9)	3 (7.1)	4 (5.9)	3 (7.1)
Median duration of response (95% CI), months	NR (7.0–NR)	NR (NR–NR)	NR (NR–NR)	NR (NR–NR)
Response duration ≥6 months, % (95% CI)	95.5 (71.9–99.4)	90.9 (50.8–98.7)	95.7 (72.9–99.4)	91.7 (53.9–98.8)
Response duration ≥12 months, % (95% CI)	83.5 (43.2–96.2)	90.9 (50.8–98.7)	95.7 (72.9–99.4)	91.7 (53.9–98.8)
Median progression-free survival (95% CI), months	13.9 (4.1–NR)	4.2 (2.2–NR)	NR (6.9–NR)	NR (2.6–NR)
6-month progression-free survival rate, % (95% CI)	60.4 (47.5–71.0)	49.8 (33.5–64.0)	64.9 (52.1–75.1)	54.8 (38.2–68.7)
12-month progression-free survival rate, % (95% CI)	55.0 (41.1–66.9)	45.9 (29.5–60.9)	62.7 (49.5–73.3)	50.9 (33.9–65.6)

CI, confidence interval; iRECIST, response criteria for cancer immunotherapy trials; IRRC, independent radiological review committee; NA, not applicable; NR, not reached; RECIST, Response Evaluation Criteria in Solid Tumors.

Supplementary Table S3. Best overall response, duration of response, and progression-free survival by IRRC and overall survival in the sensitivity analysis population

	Sensitivity analysis population (*n* = 58)	
	RECIST version 1.1	iRECIST
Objective response rate		
n (%)	24 (41.4)	24 (41.4)
95% CI	28.6–55.1	28.6–55.1
Disease control rate		
n (%)	42 (72.4)	42 (72.4)
95% CI	59.1–83.3	59.1–83.3
Complete response, *n* (%)	2 (3.4)	2 (3.4)
Partial response, *n* (%)	22 (37.9)	22 (37.9)
Stable disease, *n* (%)	18 (31.0)	18 (31.0)
Confirmed progressive disease, *n* (%)	13 (22.4)	8 (13.8)
Unconfirmed progressive disease, *n* (%)	NA	5 (8.6)
Non-evaluable, *n* (%)	3 (5.2)	3 (5.2)
Median duration of response (95% CI), months	NR (NR–NR)	NR (NR–NR)
Response duration ≥6 months, % (95% CI)	95.2 (70.7–99.3)	95.2 (70.7–99.3)
Response duration ≥12 months, % (95% CI)	95.2 (70.7–99.3)	95.2 (70.7–99.3)
Median progression-free survival (95% CI), months	NR (13.8–NR)	NR (NR–NR)
6-month progression-free survival rate, % (95% CI)	69.4 (55.4–79.7)	70.6 (56.5–80.9)
12-month progression-free survival rate, % (95% CI)	69.4 (55.4–79.7)	70.6 (56.5–80.9)
Median overall survival (95% CI), months	NR (16.0–NR)	
6-month overall survival rate, % (95% CI)	91.3 (80.4–96.3)	
12-month overall survival rate, % (95% CI)	88.8 (76.6–94.9)	

CI, confidence interval; iRECIST, response criteria for cancer immunotherapy trials; IRRC, independent radiological review committee; NA, not applicable; NR, not reached; RECIST, Response Evaluation Criteria in Solid Tumors.

Supplementary Table S4. Objective response rate, disease control rate, duration of response, and progression-free survival by IRRC per RECIST version 1.1 and overall survival across patient subgroups in the main efficacy analysis population

	Objective response rate		Disease control rate		Duration of response (month)			Progression-free survival (month)			Overall survival (months)		
	n/*N* (%)	95% CI	*n*/*N* (%)	95% CI	Median (95% CI)	Response duration ≥6 months, % (95% CI)	Response duration ≥12 months, % (95% CI)	Median	6-month progression-free survival rate, % (95% CI)	12-month progression-free survival rate, % (95% CI)	Median	6-month overall survival rate, % (95% CI)	12-month overall survival rate, % (95% CI)
PD-L1 expression													
Positive	14/30 (46.7)	28.3–65.7	20/30 (66.7)	47.2–82.7	NR (NR–NR)	100 (100–100)	100 (100–100)	NR (2.7–NR)	67.9 (47.4–81.9)	67.9 (47.4–81.9)	NR (NR–NR)	93.2 (75.5–98.3)	88.3 (67.3–96.2)
Negative	10/29 (34.5)	17.9–54.3	20/29 (69.0)	49.2–84.7	NR (2.8–NR)	90 (47.3–98.5)	NR (NR–NR)	NR (2.2–NR)	54.3 (34.5–70.5)	54.3 (34.5–70.5)	NR (10.1–NR)	82.8 (63.4–92.4)	70.6 (45.4–85.8)
Tumour mutational burden													
High	22/55 (40.0)	27.0–54.1	39/55 (70.9)	57.1–82.4	NR (NR–NR)	94.7 (68.1–99.2)	94.7 (68.1–99.2)	NR (13.8–NR)	67.6 (53.1–78.5)	67.6 (53.1–78.5)	NR (16.0–NR)	90.8 (79.4–96.1)	88.2 (75.4–94.6)
Low	3/8 (37.5)	8.5–75.5	5/8 (62.5)	24.5–91.5	NR (NR–NR)	NR (NR–NR)	NR (NR–NR)	2.4 (1.2–NR)	37.5 (8.7–67.4)	NR (NR–NR)	10.1 (1.9–NR)	75.0 (31.5–93.1)	37.5 (1.4–79.8)
Tumour type													
CRC	20/53 (37.7)	24.8–52.1			7.0 (1.6–13.1)*								
Non-CRC	6/15 (40.0)	16.3–67.7			3.5 (1.4–5.5)*								

CI, confidence interval; CRC, colorectal cancer; IRRC, independent radiological review committee; NR, not reached; PD-L1, programmed death-ligand 1; RECIST, Response Evaluation Criteria in Solid Tumors.

* Range is shown instead of 95% CI.

Supplementary Table S5. Change in EQ-5D-5L scores from baseline in the main efficacy analysis population

	Increased	Unchanged	Reduced	Missing
Mobility	19 (27.9)	41 (60.3)	3 (4.4)	5 (7.4)
Self-care	8 (11.8)	53 (77.9)	2 (2.9)	5 (7.4)
Usual activities	18 (26.5)	44 (64.7)	1 (1.5)	5 (7.4)
Pain/discomfort	20 (29.4)	32 (47.1)	11 (16.2)	5 (7.4)
Anxiety/depression	21 (30.9)	38 (55.9)	4 (5.9)	5 (7.4)
Visual analogue scale	41 (60.3)	14 (20.6)	8 (11.8)	5 (7.4)

Data are presented as *n* (%). The worst post-baseline result was used to calculate the change from baseline for each patient.

EQ-5D-5L, five-level version of the EuroQol five-dimensional questionnaire.

Supplementary Table S6. Change in EORTC QLQ-C30 scores from baseline in the main efficacy analysis population

	Increased	Unchanged	Reduced	Missing
Global health status	18 (26.5)	18 (26.5)	27 (39.7)	5 (7.4)
Functional scale				
Physical function	31 (45.6)	23 (33.8)	9 (13.2)	5 (7.4)
Role function	23 (33.8)	33 (48.5)	7 (10.3)	5 (7.4)
Emotional function	31 (45.6)	24 (35.3)	8 (11.8)	5 (7.4)
Cognitive function	23 (33.8)	34 (50.0)	6 (8.8)	5 (7.4)
Social function	22 (32.4)	33 (48.5)	8 (11.8)	5 (7.4)
Symptom scale				
Fatigue	32 (47.1)	18 (26.5)	13 (19.1)	5 (7.4)
Nausea and vomiting	15 (22.1)	35 (51.5)	13 (19.1)	5 (7.4)
Pain	24 (35.3)	24 (35.3)	15 (22.1)	5 (7.4)
Dyspnoea	20 (29.4)	39 (57.4)	4 (5.9)	5 (7.4)
Insomnia	18 (26.5)	34 (50.0)	11 (16.2)	5 (7.4)
Appetite loss	16 (23.5)	36 (52.9)	11 (16.2)	5 (7.4)
Constipation	18 (26.5)	38 (55.9)	7 (10.3)	5 (7.4)
Diarrhoea	23 (33.8)	30 (44.1)	10 (14.7)	5 (7.4)
Financial difficulties	19 (27.9)	36 (52.9)	8 (11.8)	5 (7.4)

Data are presented as *n* (%). The worst post-baseline result was used to calculate the change from baseline for each patient.

EORTC QLQ-C30, European Organisation for Research and Treatment of Cancer Quality of Life Questionnaire-Core 30.

Supplementary Table S7. Adverse drug reactions (≥5% in both analysis sets)

Adverse events	Safety set (n = 108)	Main efficacy analysis population (n = 68)
Any adverse drug reactions	86 (79.6)	58 (85.3)
Hypothyroidism	20 (18.5)	13 (19.1)
Alanine aminotransferase increased	19 (17.6)	15 (22.1)
Aspartate aminotransferase increased	19 (17.6)	12 (17.6)
Anaemia	13 (12.0)	9 (13.2)
Proteinuria	13 (12.0)	9 (13.2)
Blood bilirubin increased	10 (9.3)	9 (13.2)
White blood cell decreased	10 (9.3)	9 (13.2)
Diarrhoea	9 (8.3)	7 (10.3)
Hyperthyroidism	9 (8.3)	7 (10.3)
Gamma-glutamyltransferase increased	9 (8.3)	6 (8.8)
Neutrophil count decreased	8 (7.4)	8 (11.8)
Fatigue	7 (6.5)	5 (7.4)
Alkaline phosphatase increased	7 (6.5)	4 (5.9)
Abnormal liver function	6 (5.6)	5 (7.4)
Creatine phosphokinase increased	6 (5.6)	5 (7.4)
Blood lactate dehydrogenase increased	6 (5.6)	4 (5.9)

Data are presented as n (%).

Supplementary Table S8. Serplulimab accumulation ratio across patient subgroups in the pharmacokinetic set

	R_{Cmax}		$R_{Ctrough}$	
	n	Mean (STD)	*n*	Mean (STD)
Overall	48	2.0 (0.5)	49	4.3 (3.1)
ADA				
Positive	4	2.0 (0.7)	4	3.0 (1.5)
Negative	42	2.0 (0.5)	43	4.3 (3.2)
PD-L1 expression				
Positive	20	1.9 (0.5)	23	4.9 (4.3)
Negative	22	2.1 (0.5)	22	3.9 (1.1)
TMB				
High	35	2.0 (0.6)	36	4.6 (3.5)
Low	10	1.9 (0.4)	10	3.5 (1.6)
MSI				
MSI-H	40	2.0 (0.5)	41	4.6 (3.3)
MSS/MSI-L	8	1.9 (0.5)	8	3.1 (1.5)

ADA, antidrug antibody; MSI, microsatellite instability; MSI-H, microsatellite instability-high; MSI-L, microsatellite instability-low; MSS, microsatellite stable; PD-L1, programmed death-ligand 1; R_{Cmax}, accumulation ratio of the peak serum drug concentration; $R_{Ctrough}$, accumulation ratio of the trough serum drug concentration; STD, standard deviation; TMB, tumour mutational burden.

Supplementary Figure S1. Serum concentrations of serplulimab versus time

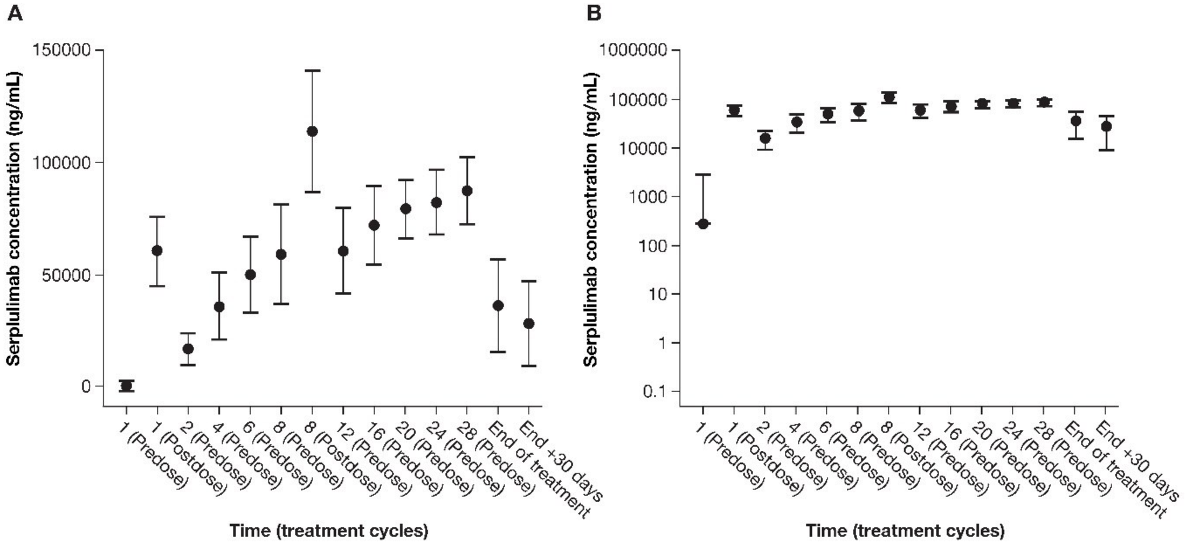

Mean serum concentration of serplulimab (predose and postdose) over time, plotted on (A) a linear scale or (B) a semi-log scale. Error bars represent standard deviation.
